# Objective assessment of lameness in cattle after foot surgery

**DOI:** 10.1371/journal.pone.0209783

**Published:** 2018-12-28

**Authors:** Lindsay L. Buisman, Maher Alsaaod, Esther Bucher, Johann Kofler, Adrian Steiner

**Affiliations:** 1 Clinic for Ruminants, Vetsuisse-Faculty, University of Berne, Berne, Switzerland; 2 Faculty of Veterinary Medicine, Utrecht University, Utrecht, The Netherlands; 3 Clinic for Ruminants, University of Veterinary Medicine Vienna, Vienna, Austria; United States Department of Agriculture, Agricultural Research Service, UNITED STATES

## Abstract

Assessment of lameness in cattle after foot surgery is important to monitor the recovery period, to improve the long-term success and the cows` welfare. This longitudinal multicenter retrospective study was carried out to evaluate the usefulness of automated tools of weight bearing and gait analysis following foot surgery to support the clinician to monitor lameness in cattle. For this purpose, the effect of involvement of different anatomical structures and the use of different surgery methods on gait parameters of post-operative recovery was assessed. The study consisted of 2 experiments and included cattle with unilateral foot pathologies located in the digital region which needed 1 (experiment 1; *n* = 30) or 2 (experiment 2; *n* = 4) surgical interventions. The surgical techniques were debridement, joint lavage, partial resection of bones, tendons or synovial structures, total resection of the sesamoid bone and digit amputation. Two accelerometers (400 Hz; kinematic outcome = stance phase duration; kinetic outcome = foot load and toe-off), a 4-scale weighing platform (difference of mean weight distribution across the limbs; Δ_weight_) and a subjective locomotion score were used to evaluate gait parameters every 3 to 4 days after surgery. A repeated measures ANOVA was used in experiment 1 and a receiver operator characteristic analysis was used to determine the optimal cutoff values in experiment 2. Results showed that the differences across limbs for the pedogram variables of stance phases and peaks of foot load and toe-off, Δ_weight_ and the locomotion score were highest if joints or sesamoid bones were involved, suggesting that these cattle were more severely lame compared to cattle with more superficial foot pathologies. There was a significantly lower degree of lameness after surgical debridement and after digit amputation compared to partial and total resection of anatomical structures of the foot. The use of accelerometers and a 4-scale weighing platform represent promising objective tools for post-operative monitoring of lameness and can support the clinician in gait assessment to improve the long-term success of surgical interventions in the area of the foot.

## Introduction

Lameness has a major impact on the welfare and health of cattle worldwide. Economic losses caused by lameness, are the result of decreased productivity, increased risk of premature culling, higher treatment costs and additional management time [1–3]. The prevalence of lameness in cattle varies considerably, and is described to be 14.8% in Switzerland [4], 36.8% in the United Kingdom [5] and up to 54.8% in the North-East of the United States [6]. Lameness is associated with pain and discomfort due to pathologies involving the locomotor system [7–9], most frequently affecting the hind limbs, and in about 90% of cases the foot [10–12].

Conservative treatment of claw disorders includes functional claw trimming, reduction of pressure on the horn defect and administration of nonsteroidal anti-inflammatory drugs (NSAIDs) or antibiotics, the latter only in cases with a justified indication. Foot surgery in cattle can be a promising treatment option if conservative treatment is not sufficient and if euthanasia or slaughter is not an option [13–17]. Most common indications for surgical treatment are non-healing lesions (e.g. white line disease, toe ulcers or sole ulcers, secondary infected by digital dermatitis-associated treponemes), acute deep injuries (e.g. perforations), and deep digital sepsis (e.g. septic arthritis of the distal interphalangeal joint, septic tenosynovitis of the digital flexor tendon sheath and osteomyelitis of the distal phalanx) [18–24]. Surgical treatment of claw disorders and deep digital sepsis include claw-preserving surgery techniques (e.g. surgical debridement of infected bone, resection of the distal sesamoid bone and resection of the distal interphalangeal joint) and various methods of amputation of the complete claw or distal digit [13,15–17,19–22,24–28]. The purpose of the surgical treatment is to use the least invasive technique possible that is appropriate for the lesion in question, in order to resolve lameness and pain and restore production [16]. The invasiveness of the surgery technique selected depends on several factors, such as the lesion type, the extent of involved anatomical structures and the diagnostic options for their exact recognition, the economic feasibility and the practicality of the surgical intervention [16].

Thorough post-operative aftercare, dry and clean housing conditions and effective pain management is essential for success of all surgical procedures [7,8,24,29]. A wooden or rubber block has to be glued on the healthy partner claw to prevent weight bearing of the operated and painful claw and to support the lesion healing [8,16,19,21,23,24]. Wound care and clinical examination of the lesion healing progress is done during regular bandage changes. Depending on the healing progress and surgeons`preferences, the first bandage change should be done 2 to 3 days after surgery, followed by further bandage changes every 3 to 4 days until the wound is completely covered with healthy granulation tissue [21,24]. The long-term success will also depend on early detection of new claw lesions and complications in the partner claw or in the claws of the contralateral limb [16,25,27]. In addition, post-operative observation is important to evaluate if another treatment or second surgery is required.

Precise post-operative evaluation of lameness is important to monitor the recovery period, to improve long-term success and the cows’ welfare [16]. Several clinical locomotion-scoring systems can be used for assessment of lameness [30,31]. However, subjective observation of lameness can be difficult for gait assessment in slightly lame cows and non-lame cows with foot pathologies [31–34]. Objective measurement of gait pattern, such as using accelerometers to measure kinetic and kinematic gait variables and using a 4-scale weighing platform to measure asymmetry of weight distribution across the limbs, has been shown a promising approach for assessment of lameness in cattle [35–39]. It can be combined with clinical gait assessment and can support the observer in assessment of lameness in cattle [40,41]. The aims of our study were (1) to evaluate the correlation between the locomotion score system and objective parameters of the cow pedogram and weighing platform, (2) to make an objective evaluation of the effect of the involvement of different anatomical structures and the use of different surgery methods for the foot on gait parameters during post-operative recovery, and (3) to evaluate the post-operative differences in gait parameters after surgical debridement in cattle undergoing one surgery compared to cattle in which a second surgery is required.

## Materials and methods

### Ethical statement

The study protocol was approved by the animal experimentation committee of the canton of Bern, Switzerland (permission # 25601) and it was discussed and approved by the institutional ethics and animal welfare committee of the University of Veterinary Medicine Vienna, Austria in accordance with GSP guidelines and national legislation (permission ETK-02/08/2017).

### Experimental procedures

#### Animals and inclusion criteria

The multicenter longitudinal retrospective study consisted of 2 experiments and was carried out between December 2015 and April 2018 at the Clinic for Ruminants, Vetsuisse-Faculty, University of Bern, Switzerland and at the Clinic for Ruminants, University of Veterinary Medicine Vienna, Austria. Cattle (*n* = 34) with unilateral foot pathologies located in the area of the foot (up to and including the fetlock joint), that had been referred to the clinic for further evaluation, were included. Diagnosis was based on a thorough clinical and orthopaedic examination, diagnostic claw trimming, ultrasonography, radiography and synoviocentesis, if indicated [12,42,43]. Foot pathologies were classified according to the ICAR Claw Health Atlas (http://www.icar.org/Documents/ICAR_Claw_Health_Atlas.pdf). Data retained from medical records included age, weight, breed, duration of hospitalization, clinical findings, surgery methods and whether a second surgery was needed.

Experiment 1 included 30 cattle which needed 1 surgical treatment. The mean (±SD) age was 59.67 months (±35.34), with a mean body weight of 604.86 kg (±117.61), a mean days in milk of 152.65 days (±74.33) and a mean daily milk yield of 19.58 L (±10.25). The mean period of stay at the farm animal clinics of Bern and Vienna was 16.43 days (±9.16). The breeds involved were Holstein Friesian (n = 8), Red Holstein (n = 2), Braunvieh (n = 1), Fleckvieh (n = 13), Simmental (n = 1), Eringer (n = 3), Pustertaler Sprinze Bull (n = 1) and Montbéliard (n = 1).

Experiment 2 included 4 cattle which needed 2 surgical interventions. The mean (±SD) age was 40.5 months (±23.53), with a mean body weight of 536.0 kg (±241.16), a mean days in milk of 129.0 days (±185.33) and a mean daily milk yield of 42.5 L (±10.61). The mean period of stay at the farm animal clinic was 34.75 days (±16.10). The breeds involved were Holstein Friesian (n = 2) and Fleckvieh (n = 2). Table 1 lists the pathological findings and used surgery methods in cattle of experiments 1 and 2. The full description of the used cattle can be found in S1 Table. Indications for a second surgical intervention were (i) no remarkable improvement of the locomotion score within the period until 2^nd^ surgery, (ii) no improvement of wound healing (e.g. development of new purulent exudate, increasing swelling and inflammation) and (iii) persistent signs of an active infectious process present during the follow-up period after first surgery.

**Table 1:** Pathological findings and used surgery methods in cattle of experiment 1 (cattle with 1 surgical treatment; *n* = 30) and experiment 2 (cattle with 2 surgical treatments; *n* = 4). The clinical examination, pathological findings and used surgery methods were used as the gold standard for further group allocation.

	Experiment 1 (*n* = 30)	Experiment 2 (*n* = 4)
Pathological findings^1^	SU (*n =* 8), BA (*n* = 1), DS (*n* = 4), HF (*n* = 3), WLA (*n* = 3), WLF (*n* = 6), IH (*n* = 2), IP (*n* = 1), coronet phlegmon (*n =* 1), bulb phlegmon (*n =* 1), TS (*n =* 1), TN (*n* = 1), septic serous arthritis of DIJ (*n =* 2), septic (necrotic) arthritis of DIJ (*n =* 7), septic serous arthritis of PIJ (*n =* 1), septic (sero-fibrinous) tenovaginitis of CDFTS (*n =* 10), septic (necrotic) tenovaginitis of DFTS (*n* = 3), septic osteitis of P3 (*n* = 8), osteomyelitis of P3 (*n =* 1), osteolysis of P3 (*n =* 1), osteolysis of P2 (*n =* 1), osteolysis of the sesamoid bone (*n =* 1), necrotic DDFT (*n =* 3), necrotic flexor tubercle (*n =* 4), and necrotic sesamoid bone (*n =* 4).	SU (*n* = 2), SA (*n* = 2), DS (*n* = 1), WLF (*n* = 1), IH (*n* = 2), septic osteitis of P3 (*n* = 1), osteomyelitis of P1 (*n* = 1), septic arthritis of DIJ (*n* = 1), osteoarthritis of DIJ (*n* = 1), and septic fibrinous arthritis of MTPJ (*n* = 1).
Involved anatomical structures^1^	P3 (*n =* 11), P2 (*n =* 1), CDFTS (*n* = 12), DDFT (*n =* 12), SDFT (*n =* 10), DIJ (*n =* 9), PIJ (*n =* 1), sesamoid bone (*n* = 6), and flexor tubercle of P3 (*n =* 4).	P3 (*n* = 4), P2 (*n = 2*), P1 (*n* = 1), DIJ (*n* = 2), PIJ (*n = 1*), MTPJ (*n* = 1), and sesamoid bone (*n* = 1).
Used surgical methods^2^	Surgical debridement, opening and flushing CDFTS, partial resection SDFT, partial resection DDFT, resection WLD, partial resection toe tip, partial resection flexor tubercle, total resection sesamoid bone, digit amputation (disarticulation through PIJ), digit amputation (through P2), and digit amputation (through P1).	Surgical debridement, lavage DIJ, partial resection DIJ, partial resection SDFT, partial resection DDFT, and digit amputation (through P1).

^1^In each cow, one or more unilateral foot pathologies were diagnosed, in which one or more anatomical structures were involved.

^2^In each cow, one or more surgical methods were used.

(SU) Sole ulcer

(SA) Sole abscess

(BA) Bulb abscess

(DS) Double sole

(TS) Thin sole

(HF) Horn fissure

(IH) Interdigital hyperplasia

(IP) Interdigital phlegmon

(TN) Toe necrosis

(WLA) White-line abscess

(WLF) White-line fissure

(WLD) White-line disease

(P1) Proximal phalanx

(P2) Middle phalanx

(P3) Distal phalanx

(DIJ) Distal interphalangeal joint

(PIJ) Proximal interphalangeal joint

(MTPJ) Metatarsophalangeal joint

(CDFTS) Common digital flexor tendon sheath

(DDFT) Deep digital flexor tendon

(SDFT) Superficial digital flexor tendon

#### Allocation of cattle to experimental groups

In each cow, one or more unilateral foot pathologies were diagnosed, in which one or more anatomical structures were affected and one or more surgical methods were used. Each cow of experiments 1 and 2 was allocated to a location [**LOC**] and a surgery [**SURG**] group. LOC allocation was based on the location of the foot pathology, the deepness of the infection or injury, non-perforating or perforating the claw capsule and the extent of involved anatomical structures. SURG allocation was based on the type of surgical treatment that was used.

Cattle with non-perforating foot pathologies were allocated to group **LOC1** (experiment 1: *n* = 9). Non-perforating foot pathologies are superficial foot pathologies without perforation of the corium and subcutis and without involvement of inner structures of the claw [18–21,24]. The other LOC groups included cattle with perforating foot pathologies in which the corium and subcutis were perforated and inner structures of the claw were involved. **LOC2** (experiment 1: *n* = 7 and experiment 2: *n* = 2) included cattle with involvement of the distal phalanx alone. **LOC3** (experiment 1: *n* = 5) included cattle with involvement of the superficial digital flexor tendon, deep digital flexor tendon or common digital flexor tendon sheath alone, or in combination with adjacent or more superficial anatomical structures, but without involvement of joints or sesamoid bones. Finally, **LOC4** (experiment 1: *n* = 9 and experiment 2: *n* = 2) included cattle with involvement of at least joints, sesamoid bones and associated bursae alone, or in combination with adjacent or more superficial anatomical structures.

Cattle with only surgical debridement or joint lavage as surgical treatment were allocated to group **SURG1** (experiment 1: *n* = 15 and experiment 2: *n* = 5). Surgical debridement is a technique with the use of instruments, such as a scapel, claw knife, curette or hammer and chisel, to manually remove altered, infected or necrotic tissue and debris [15,21]. Corrective claw trimming is often combined with surgical debridement and is used to remove altered, loose and undermined claw horn tissue that may cause further injury to the corium and to transfer the weight to the healthy claw in order to reduce the weight bearing of the diseased claw [19,44]. Joint lavage includes flushing, cleaning and ridding out of debris, microorganisms, inflammation products and fibrin by using an isotonic saline solution, in which the function and morphology of the joint is preserved [45,46]. **SURG2** (experiment 1: *n* = 6 and experiment 2: *n* = 2) included cattle with partial resection of the superficial digital flexor tendon, deep digital flexor tendon, distal interphalangeal joint, flexor tubercle or toe tip alone or as a part of the surgical treatment, but without total resection or digit amputation. Partial resection is a claw-preserving surgery technique, in which altered, damaged, necrotic or infected anatomical structures and tissues (e.g. parts of bones and tendons, bony or cartilaginous parts of joints) partially are removed [15,19]. **SURG3** (experiment 1: *n* = 3) included cattle with total resection of the sesamoid bone alone or as part of the surgical treatment, but without digit amputation. Total resection is a claw-preserving surgery technique, in which altered, damaged, necrotic or infected anatomical structures or tissues completely are removed [15,19]. Finally, cattle with digit amputation alone or as a part of the surgical treatment were allocated to group **SURG4** (experiment 1: *n* = 6 and experiment 2: *n* = 1). Digit amputation is the surgical removal of the digit at a joint by disarticulation or by severing a bone. Several surgical amputation techniques are described [15–17,19–21,24,28]. Low amputation is the removal of the digit at the level of the distal interphalangeal joint. High amputation is the removal of the digit at the level of the middle of P2, the proximal interphalangeal joint, or the at the distal aspect of P1. The coronary band may or may not be preserved. If indicated, the skin flaps may be preserved and sutured to cover the amputation surface [15,16,19,24].

#### Pre- and post-operative surgical procedures

Surgeries were performed while the cow was lying on a hydraulic tilt table in lateral recumbency. Penicillin G sodium (30,000 IU/kg IV) and ketoprofen (3 mg/kg IV) were administered before surgery, in which penicillin was only administered in case of lesions with bone involvement. Analgesia was performed with retrograde intravenous local anesthetic (20 mL 2%-Lidocaine), as described by Antalovsky [47] and Steiner et al. [48]. If necessary, xylazine (0.2 mg/kg IM) was administered for sedation to relieve stress and keep the cow calm on the operating table [8,16,24,29,49]. Corrective claw trimming was done before surgery. If indicated, a wooden or rubber block was glued to the sole of the healthy partner claw after surgery [18–21]. Depending on the degree of infection, extent of involved anatomical structures and progress of lesion healing, post-operative care included systemic administration of penicillin G sodium (30,000 IU/kg IV TID), oxytetracycline (10 mg/kg IV BID) or gentamycin (6.6 mg/kg IV SID for 3 days always combined with penicillin G sodium) for 3 to 20 days, and systemic administration of ketoprofen (3 mg/kg IV SID) for 1 to 2 days continued with oral administration of ketoprofen (4 mg/kg PO) for 5 to 15 days. During the post-operative period, cattle were housed at the farm animal clinics of Bern and Vienna in a clean and dry environment. The following health parameters were recorded at daily post-operative clinical examination: posture, general behavior, rectal body temperature, heart rate, respiratory rate, rumen fill, rumen motility, succussion and percussion auscultation, abdominal shape, and appearance and amount of feces. Post-operative clinical examination of the claws focused on lameness, lesion healing, complications, the health of the partner claw and the health of the claws of the opposite limb. This examination was performed in a claw trimming chute or on a hydraulic tilt table and was done during regular bandage (semi-occlusive, nonadherent) changing, which was every 2 to 3 days after surgery. A bandage was applied until the wound was completely covered with healthy granulation tissue and epithelialization had occurred.

### Data collection

#### Recording and analysis of gait variables

On each experimental day, weight distribution among limbs was measured while cattle were standing on a 4-scale weighing platform (1.94 x 1.06 m; ITIN & HOCH GmbH, Fütterungstechnik). The weighing platform was only available for the cows at the farm animal clinic in Bern (*n* = 23). The platform consisted of 4 recording units (0.78 x 0.55 m), as described by Nechanitzky et al. [37]. The weight of each limb delivered to the respective unit was recorded for 5 min with a frequency of 10 Hz. The mean weight and standard deviation (**SD**) applied on each limb were calculated for each 5 min measurement.

Before data recording of gait kinetics and kinematics, cattle were equipped with 2 stand-alone 3-dimensional accelerometers (400 Hz; USB Accelerometer X16-4, Gulf Coast Data Concept, Waveland, MS), which were fitted at the level of the mid metatarsus/metacarpus to both hind or fore limbs of the affected limb pair as described by Alsaaod et al. [38]. The validated Cow-Gait-Analyzer (University of Bern, Switzerland) was used to analyze the gait cycle variables as described by Alsaaod et al. [50]. The pedogram parameters included the temporal event (kinematic outcome) relative stance phase duration and the peaks (kinetic outcome) foot load and toe-off. Table 2 lists the gait cycle variables and definitions used in this study.

**Table 2:** Definitions of the cows’ gait variables including the kinematic (temporal) and kinetic (peak) pedogram variables at the level of the metatarsi extracted by the use of the Cow-Gait-Analyzer as described by Alsaaod et al. [38,50] and the 4-scale weighing platform variables [37].

Method	Item	Gait variable	Definition
Cow pedogram	Kinetic (temporal)	Stance phase (%)	Percentage proportion of time that the claw is in contact with the ground to the total gait cycle duration (interval between foot-load peak and consecutive toe-off peak)
	Kinematic (peak)	Foot load (g)	Maximum acceleration (peak) of the initial ground contact of the claw
		Toe-off (g)	Maximum acceleration (peak) of the termination of the ground contact of the tip of the claw
4-scale weighing platform	Kinetic (temporal)	Mean weight (kg)	Mean weight applied on each limb
		SD_weight_ (kg)	Standard deviation of the weight applied on each limb
		Δ_weight (%)_	The mean weight difference (%) across the healthy and the lame limb within the affected limb pair

#### Clinical locomotion score assessment

All cattle were videotaped using a digital video camera (50 frames/s, Sony HDR-PJ740VE, Sony Corporation, Tokyo, Japan; 50 frames/s, Samsung Galaxy XCover GT-S7710, Seoul, South Korea) to record the locomotion while a trained animal caretaker guided the cattle with a halter and encouraged them to walk in a straight line and an eight figure on an asphalt floor. The video recordings were blinded as to group allocation and scored using a 1 to 5 numerical rating system (NRS) with 0.5-point increments (where 1 = non-lame and 5 = severely lame). This score was based on 6 specific gait characteristics (back arch, head bob, tracking up, joint flexion, asymmetric steps and reluctance to bear weight; as described by Flower and Weary [31]). Each video recording was independently scored by 2 trained observers (LB; MA). The recordings were scored in random order, in order to blind observers. Recordings with scores deviating for more than 1 point among the 2 observers were independently evaluated once again. A few recordings with scoring results deviating for more than 1 point after second rating were rated together and discussed until agreement of a difference of 1 point or less was reached. The mean value for each measurement was calculated and used for further analysis for the specific animal.

#### Recording period

The locomotion score, weight distribution and gait kinetics and kinematics were measured before and after surgery. The basic measurement (day -1) was directly prior to surgery (where surgery is day = 0). The other measurements were during the post-operative period at the farm animal clinic every 3 to 4 days after surgery, starting at day +1 and continuing with day +4, +7, +10, and +13 relative to surgery. The measurements were always before the routine bandage change. In experiment 2, the last measurement before the second surgery was also used as second basic measurement. The clinical case management software package POLYPOINT (POLYPOINT, Berlin, Germany) was used to collect information about the history, productivity and clinical examinations of each cow.

### Data analysis and statistics

The kinetic and kinematic pedogram variables were calculated as the absolute difference across the limbs at the level of the metatarsi (**Δ**_**MT**_), in which toe-off and foot load $(\text{acc})=ABS\;\left(\frac{healthy\;\text{lim}b-lame\;limb}{1024}\right)$ and relative stance phase duration (%) = *ABS (healthy limb − lame limb) x* 100%. The weight distribution was calculated as the percentage absolute difference of the mean weight across the limbs $\left(\Delta_{weight}\;\left(\%\right)=\frac{mean\;weith\;healthy\;limb-mean\;weight\;lame\;limb}{mean\;weight\;lame\;limb+mean\;weight\;healthy\;limb}\;x\;100\%\right)$.

All statistical analyses were performed using the software package NCSS (NCSS, LCC, Kaysville, UT) (S1 and S2 Datasets). The normality of all analysed variables was checked with the Shapiro-Wilk test. Most of the variables were not normally distributed. Therefore, a natural logarithm was calculated, and the transformed variables were used for further analysis (Shapiro W test statistic for all variables was > 0.95). The agreement between the locomotion score and the objective parameters of the cow pedogram and weighing platform was determined using correlation coefficient. The variables were not normally distributed; therefore, Spearman nonparametric correlation coefficient was used for the analysis. A correlation coefficient (r_s_) of r_s_ = ≥ 0.9 was rated as very high, r_s_ = 0.68 to 1.0 as strong or high, r_s_ = 0.36 to 0.67 as moderate, and r_s_ = ≤ 0.35 as weak correlation [51].

In experiment 1, a repeated measures ANOVA was performed to evaluate the effect of involvement of different anatomical structures (LOC) on gait parameters of post-operative recovery (**part I**), and to evaluate the different surgery methods (SURG) for the foot on gait parameters of post-operative recovery (**part II**). The cow was set as a subject variable with LOC and SURG as between factor variable (fixed effect) and time point relative to surgery as within factor variable (fixed effect). All basic measurements were excluded, as it was shown in experiment 1 that there were cows with only a block after surgery but without a block during the basic measurement (day -1). The group distribution and relationship between the involved anatomical structures and the used surgery methods was determined with the Pearson`s Chi-Square Test (contingency tables). In experiment 2, a repeated measures ANOVA was performed to evaluate the post-operative differences in gait parameters for surgical debridement in cattle undergoing 1 surgery (experiment 1) compared to cattle undergoing 2 surgeries (experiment 2). A receiver operator characteristic (ROC)-analysis was used to calculate the optimal cut-off values for the different gait cycle variables for identifying whether a cow needed a second surgery. Bonferroni corrected *P*-value was calculated to account for multiple comparisons. The significance probability was set at *P* ≤ 0.05 (without Bonferroni adjustment).

## Results

### Correlations between parameters

There was a moderate correlation between the locomotion score and the weighing platform parameter Δ_weight_ (0.60) and pedogram parameters Δ_MT_ of relative stance phase duration, toe-off and foot load (0.65, 0.61 and 0.45, respectively). The correlation between the weighing platform parameter Δ_weight_ and the pedogram parameters Δ_MT_ of foot load, toe-off and relative stance phase duration was also moderate (0.54, 0.50 and 0.45, respectively).

### Experiment 1

#### Part I

Mean (SEM) differences across limbs of the various gait variables during the post-operative period (d +1, +4, +7, +10, and +13) for different involved anatomical structures are given in Table 3. LOC4 revealed significantly higher differences across limbs for variables of Δ_MT_, Δ_weight_ and the locomotion score compared to LOC1 (*P <* 0.05). Moreover, differences across limbs for the gait variables Δ_MT_ of relative stance phase duration and toe-off were significantly higher for LOC4 compared to LOC2. Only Δ_weight_ was found to be significantly different between LOC3 and LOC4. The gait variables Δ_MT_ of relative stance phase duration and toe-off showed significantly higher differences for LOC3 compared to LOC1. Furthermore, LOC3 revealed significantly higher differences for Δ_MT_ of relative stance phase duration compared to LOC2. Only Δ_weight_ was found to be significantly different between LOC1 and LOC2.

**Table 3:** Post-operative mean (SEM) differences across the limbs of an affected limb pair at the level of the metatarsi for kinematic (temporal) and kinetic (peak) pedogram parameters (Δ_MT_), weighing platform parameters (Δ_weight_ and SD_weight_) and locomotion score assessment of various involved anatomical structures (part I) in cattle of experiment 1.

Item	Variable	Experiment 1, part I (*n* = 30)								
		LOC1^1^ (*n* = 9)		LOC2^2^ (*n* = 7)		LOC3^3^ (*n* = 5)		LOC4^4^ (*n* = 9)		*P*—Value
		Mean	SEM^5^	Mean	SEM	Mean	SEM	Mean	SEM	
Pedogram kinematic (temporal)	Relative stance phase duration (%)	1.99^b^	1.20	3.21^b^	1.21	7.76^a^	1.25	8.85^a^	1.19	< 0.001*
Pedogram kinetic (peak)	Foot load (g)	1.67^b^	1.20	3.63^a^	1.21	3.49^ab^	1.25	7.30^a^	1.19	< 0.001*
	Toe-off (g)	0.87^c^	1.23	1.13^bc^	1.24	2.73^ab^	1.29	4.33^a^	1.21	< 0.001*
Weighing platform	Δ_weight_ (%)	35.67^c^	4.05	66.73^ab^	4.18	49.85^bc^	5.82	83.28^a^	10.08	< 0.001*
	SD_weight_^1^	1.03	1.06	1.18	1.06	1.34	1.09	1.02	1.15	< 0.076
Subjective method	Locomotion score	2.80^b^	1.03	2.98^ab^	1.03	3.14^ab^	1.04	3.25^a^	1.03	< 0.033*

^a-d^Means with different superscripts within rows differ significantly (*P* < 0.05).

^1^LOC1: superficial foot pathologies without perforation of the subcutis and corium, and without involvement of inner structures of the claw.

^2^LOC2: perforating deep injuries or deep digital sepsis of the foot with involvement of the distal phalanx alone.

^3^LOC3: perforating deep injuries or deep digital sepsis of the foot with involvement of the superficial digital flexor tendon, deep digital flexor tendon or common digital flexor tendon sheath alone, or in combination with adjacent or more superficial structures.

^4^LOC4: perforating deep injuries or deep digital sepsis of the foot with involvement of sesamoid bones or joints alone, or in combination with adjacent or more superficial anatomical structures.

^5^SEM: standard error of mean

*Probability level (*P*-value) is significant at alpha = 0.05

#### Part II

Mean (SEM) differences across limbs of the various gait variables during the post-operative period (d +1, +4, +7, +10, and +13) for different surgery methods are given in Table 4. SURG1 revealed significantly lower post-operative differences across limbs for Δ_weight_ and all gait variables of Δ_MT_ compared to SURG2, SURG3 and SURG4 (*P <* 0.05). The locomotion score was significantly lower after SURG1 compared to SURG2 and SURG3 (*P <* 0.05). There was no significant difference found between SURG2 and SURG3. In general, SURG3 revealed higher differences across limbs for all gait variables of Δ_MT_, Δ_weight_ and the locomotion score compared to SURG4. However, only Δ_MT_ of relative stance phase duration after SURG3 showed significantly higher differences across limbs as compared to SURG4 (*P <* 0.05).

**Table 4:** Post-operative mean (SEM) differences across the limbs of an affected limb pair at the level of the metatarsi for kinematic (temporal) and kinetic (peak) pedogram parameters (Δ_MT_), weighing platform parameters (Δ_weight_ and SD_weight_) and locomotion score assessment of the used surgery methods (part II) in cattle of experiment 1.

Item	Variable	Experiment 1, part II (*n* = 30)								
		SURG1^1^ (*n* = 15)		SURG2^2^ (*n* = 6)		SURG3^3^ (*n* = 3)		SURG4^4^ (*n* = 6)		*P*—Value
		Mean	SEM^5^	Mean	SEM	Mean	SEM	Mean	SEM	
Pedogram kinematic (temporal)	Relative stance phase duration (%)	2.29^c^	1.13	8.43^ab^	1.20	19.15^a^	1.29	5.62^b^	1.22	< 0.001*
Pedogram kinetic (peak)	Foot load (g)	1.80^b^	1.13	5.91^a^	1.20	7.82^a^	1.29	7.12^a^	1.22	< 0.001*
	Toe-off (g)	0.97^b^	1.17	2.46^a^	1.28	4.65^a^	1.40	4.20^a^	1.30	< 0.001*
Weighing platform	Δ_weight_ (%)	46.08^b^	3.24	65.56^a^	5.56	83.28^a^	10.83	-	-	< 0.001*
	SD_weight_^1^	1.11	1.04	1.30	1.07	1.02	1.15	-	-	< 0.123
Subjective method	Locomotion score	2.81^b^	1.02	3.23^a^	1.03	3.55^a^	1.05	3.10^ab^	1.04	< 0.001*

^a-d^Means within a row with different superscripts differ significantly (*P* < 0.05).

^1^SURG1: surgical debridement or joint lavage as surgical treatment.

^2^SURG2: partial resection alone or as part of the surgical treatment, but without total resection or digit amputation.

^3^SURG3: total resection of the sesamoid bone alone or as part of the surgical treatment, but without digit amputation.

^4^SURG4: digit amputation alone or as part of the surgical treatment.

^5^SEM: standard error of mean.

*Probability level (*P*-value) is significant at alpha = 0.05

### Surgical debridement comparison in experiment 1 and 2

The post-operative period of surgical debridement in cattle that required a second surgery revealed significantly higher differences across limbs for all gait variables of Δ_MT_, Δ_weight_ and the locomotion score compared to the post-operative period of surgical debridement in cattle which needed only 1 surgery (*P <* 0.05; Table 5). The optimal cutoff values for identifying if a cow needs a second surgery are given in Table 5.

**Table 5:** Post-operative comparison of surgical debridement in cattle which needed only 1 surgical treatment (Experiment 1, Group SURG1; *n* = 13) and the first surgery of cattle which needed 2 surgical treatments (Experiment 2, 1^st^ Surgery, Group SURG1; *n* = 3).

Item	Variable	Post-operative surgical debridement comparison (experiment 1 and 2)							
		Experiment 1 SURG1^1^ (*n* = 15)		Experiment 2 1^st^ Surgery, SURG1 (*n* = 3)		Overall			
		Mean	SEM^2^	Mean	SEM	*P*—Value	Cutoff value	Sensitivity (%)	Specificity (%)
Pedogram kinematic (temporal)	Relative stance phase duration (%)	2.29^b^	1.15	9.58^a^	1.36	< 0.001*	8.87	66.7	94.6
Pedogram kinetic (peak)	Foot load (g)	1.80^b^	1.17	5.38^a^	1.40	< 0.009*	7.47	66.7	85.7
	Toe-off (g)	0.97^b^	1.17	5.44^a^	1.40	< 0.001*	2.66	83.3	80.4
Weighing platform	Δ_weight_ (%)	46.08^b^	3.17	62.73^a^	6.86	< 0.048*	51.73	75.0	66.1
	SD_weight_	1.11^a^	1.04	1.08^a^	1.09	0.085	1.05	50.0	58.9
Subjective method	Locomotion score	2.81^b^	1.02	3.19^a^	1.04	< 0.014*	3.25	58.3	83.9

^a,b^Means with different superscripts within rows differ significantly (*P* < 0.05).

^1^SURG1: surgical debridement or joint lavage as surgical treatment.

^2^SEM: standard error of mean.

*Probability level (*P*-value) is significant at alpha = 0.05

## Discussion

The results of our study show that if joints or sesamoid bones were involved in unilateral foot pathologies, there were higher post-operative differences across limbs for all pedogram variables of stance phase and peaks of foot load and toe-off, Δ_weight_ and the locomotion score as compared to cattle with involvement of more superficial anatomical structures. When surgical debridement was chosen as surgery technique, post-operative differences across limbs were significantly lower for the pedogram variables of stance phase and peaks of foot load and toe-off, Δ_weight_ and the locomotion score as compared to the more invasive surgery methods partial resection, total resection of the sesamoid bone and digit amputation.

We combined the locomotion score, cow pedogram and 4-scale weighing platform to monitor the gait pattern during the post-operative period after foot surgery. The cow pedogram and 4-scale weighing platform are both described as promising objective tools for detection of lameness in cattle [35–39]. Alsaaod et al. [38] assessed that the cow pedogram was highly accurate to detect unilateral hind limb lameness and foot pathologies in dairy cows. Other studies reported the weighing platform as a sensitive technique for detection of lame cows, in which cows with unilateral foot pathologies had a reduced weight bearing of the affected limb and an asymmetric weight distribution across the limbs [35–37,39,52,53]. Moreover, researchers reported that lame cows showed a greater variability (SD) in weight over time applied on each limb compared to non-lame cows [39,54]. Clinical locomotion scoring systems are the current gold standard for lameness assessment in cattle [30,31]. However, these scoring systems are subjective and may not be sensitive enough to detect slightly lame cows or non-lame cows with foot pathologies [31,32,34]. Furthermore, the locomotion scoring systems require observer training, as it was shown that there is a high variation in inter- and intraobserver agreement and reliability [33,55–57]. Combining the locomotion scoring systems with objective measurement methods could be helpful for more accurate assessment of lameness in cattle [41]. We expected high correlations between these methods. However, we found only moderate correlations between the subjective locomotion score and the objective methods and between the weighing platform and cow pedogram. This moderate correlation could be explained by the use of different measurement methods, in which the weighing platform measures the lameness while a cow is standing, whereas the pedogram measurement and locomotion scoring is done during walking. Earlier studies described that cows with unilateral foot lesions causing severe lameness (LS 4–5) constantly lifted the affected limb and constantly transferred the weight to the contralateral limb while standing to relieve pain [35,37]. In contrast, cows with LS 3–4 showed a reduced weight bearing of the affected limb during walking as well, but often still used that limb to some degree in walking [31]. The tie-stall lameness scoring system is a valid method for assessment of lameness in cattle kept in tie-stalls [58]. However, we performed the locomotion score system as this system is more sensitive to detect subtle gait changes [31,58]. Furthermore, the study included pathologies of hind as well as front limbs. The stall lameness scoring system is feasible for detection of hindlimb pathologies only [58].

Earlier studies described that the weighing platform may be not accurate enough for lameness detection in the front limbs [39,53]. Moreover, cows with foot pathologies in a hind limb rarely transfer weight bearing to the front limbs, whereas cows with foot pathologies in a front limb may transfer some weight to the hind limbs to relieve pressure on the painful limbs [54,59].

We did separate analyses for LOC and SURG, as a significantly different distribution of the cattle across these groups was evident. The analysis for LOC was based on the type and deepness of the foot pathology and the anatomical structures that were involved. The analysis for SURG was based on the selected surgical technique that was necessary to resolve the lameness and pain. However, some foot pathologies can be treated with different surgery techniques. The selection of claw-preserving or amputation techniques for each foot pathology depends on several factors, such as the health and production status of the cow, the lesion type, the extent of inflammatory and necrotic alternations of the affected claw, the diagnostic options, the economic feasibility and the surgeons’ and owners’ preferences [16,24]. Surgical debridement is often used for more superficial claw disorders, whereas resection is indicated for deep digital sepsis, such as septic tenosynovitis, septic arthritis of the distal interphalangeal joint and osteomyelitis of the distal sesamoid bone [16,19,20,24,60]. Digit amputation is most commonly indicated for more severe deep digital sepsis, including severe purulent necrotising arthritis of the distal or proximal interphalangeal joint, severe osteolysis and osteomyelitis of the middle and distal phalanx, tumours and severe traumatic disorders of the claw region [14–16,18–20,24–27,60]. Neverthless, a good diagnostic work-up is important for the surgeron to decide more precisely whether a more invasive technique is indicated [24].

The SURG groups were classified depending on the surgery method selected and the invasiveness of these methods in which digit amputation is more invasive compared to total resection of the sesamoid bone, partial resection, surgical debridement and joint lavage [16,19,25]. We classified the LOC groups according to the location and deepness of the foot injury or infection, the extent of anatomical structures involved and whether it is a non-perforating or perforating foot pathology. Superficial claw disorders often have localised horn defects and only a damaged corium. Deep digital sepsis can result from perforation of the corium and subcutis, in which the localised purulent processes of the claw corium can spread to deeper anatomical structures [16,24]. Subsequently, the process spreads to the distal phalanx, the insertion of the deep digital flexor tendon, the podotrochlear bursa, the distal sesamoid bone, and finally to the distal interphalangeal joint or the digital flexor tendon sheath [15,16,20,24,27].

The difference across limbs after surgery was significantly higher for the pedogram variables of stance phase and peaks of foot load and toe-off, Δ_weight_ and the locomotion score if at least joints or sesamoid bones were involved as compared to cattle with less deep involvement of anatomical structures. This suggests that involvement of at least joints or sesamoid bones results in more severe post-operative lameness signs. This is in agreement with our clinical expectations, as the clinical signs of claw disorders and deep digital sepsis vary with the function of the anatomical structures involved in the process and the chronicity of the disease [18,61]. Cattle with superficial foot pathologies, such as interdigital phlegmon, sole ulcers and white line disease, show signs of a moderate degree of lameness, whereas deep digital sepsis results in progressively severe lameness [20,24].

The results revealed that surgical debridement had significantly lower post-operative differences across limbs for the pedogram variables of stance phase and peaks of foot load and toe-off, Δ_weight_ and the locomotion score compared to more invasive surgery methods. This suggests that the degree of lameness is the lowest after surgical debridement, which is in agreement with our expectations. Surgical debridement is a technique in which a variety of instruments (e.g. claw knife, curette and chisel) are used to manually remove altered, damaged, infected or necrotic tissue and debris to improve the healing potential of the remaining healthy tissue [15,19,61]. Surgical debridement is often indicated as treatment technique for non-perforating foot pathologies and perforating superficial foot pathologies [19,20,61]. The post-operative lameness degree after a less invasive surgery technique and a more superficial foot pathology is expected to be lower as compared to the more invasive treatment techniques for deep digital sepsis. To the best of our knowledge, there is not much literature available about surgical debridement as treatment option in cattle.

In general, this study suggested that the post-operative degree of lameness is lower after digit amputation compared to partial and total resection of anatomical structures of the foot. This is in agreement with Starke et al. [25], who showed a significant lower degree of lameness after digit amputation compared to resection of the distal interphalangeal joint. Furthermore, Starke et al. [25] reported that the degree of lameness decreases faster in cattle that have had digit amputation than those that have had resection of the distal interphalangeal joint. Joeng [62] compared resection of the distal sesamoid bone with digit amputation and found similar findings. Generally, a distinct improvement in the degree of lameness is observed 1 day after digit amputation and lameness decreases rapidly until 2 weeks after surgery [16,25]. In contrast, using claw-preserving surgery methods shows a deterioration of the lameness during the first few days after surgery [25,28,62], and lameness scores often remain high for 2–3 weeks post-operatively [16,25,28].

However, a distinct improvement of locomotion score and gait cycle variables during the first 2 weeks after digit amputation or resection was not shown in our study. The small number of cattle in each surgery group and at each time point, the exclusion of the basic measurements, the use of two different objective measurement methods and the lower sensitivity of the subjective locomotion scoring systems are possible reasons that a reliable interpretation of the post-operative improvement of the lameness was difficult in our study. Moreover, the lesion type before the surgical intervention can also influence the post-operative healing process and lameness degree. Furthermore, some claw disorders (e.g. sole ulcers and white line disease) are chronic progressive claw disorders [7], in which chronic pain results in hyperalgesia that cannot be controlled with analgesics [7,8,24,63] and can cause a delay in lameness improvement. Post-operative complications or new claw disorders at the operated claw, the partner claw or the claws of the contralateral limb can also result in a delayed post-operative recovery [14,16,24,27].

The decision to proceed with a second surgical intervention is based on several factors, such as the status of wound healing and the locomotion score. If there is no improvement of the locomotion score after surgery, it could be useful if objective tools can support the veterinarian in determining if a cow needs a second surgery. In our study, there was 1 cow which needed digit amputation after partial resection of the distal interphalangeal joint. 3 cattle needed a second surgery after surgical debridement. In case of surgical debridement, the 3 cattle that required a second surgery showed a significant higher degree of lameness during the post-operative period after the first surgery as compared to cattle that needed only 1 surgery. We calculated the optimal cutoff values for all gait parameters to identify at an early stage if a cow needs a second surgery. However, the sensitivity and specificity were not so high. The small number of cattle with surgical debridement as treatment that needed a second surgery in combination with a high standard deviation may explain the low sensitivity and specificity in our study. Therefore, larger-scale studies are needed to make a more reliable interpretation of these cutoff values.

## Conclusions

The results of this study show that cattle with unilateral deep digital sepsis are more severely lame during the recovery period after foot surgery as compared to cattle with more superficial foot pathologies. Using surgical debridement as surgery technique results in a lower degree of lameness during the post-operative recovery period as compared to the use of more invasive surgery techniques. Furthermore, cattle showed a lower degree of lameness after digit amputation compared to partial or total resection. Cutoff values for gait parameters after surgical debridement may be useful to determine at an early stage if a second surgery is required. Nevertheless, it is important to keep in mind that the severity of the foot lesion before surgery will dictate the appropriate surgery method and the degree of lameness in the recovery period. The cow pedogram and 4-scale weighing platform seem to be promising tools for post-operative monitoring of lameness and foot pathologies. These objective tools are useful to support the clinician to monitor lameness during the recovery period to improve the long-term success of surgical treatments.

## Supporting information

S1 Dataset Statistical analysis excel data. Data used for statistics.
(XLSX)

S2 Dataset Statistical analysis NCSS. Data used for statistics.
(NCSS)

S1 Table Cow description. Table of all animals and cases included in the study.
(XLSX)
